# Complete Genome Sequences of Three Rabbit Endogenous Lentivirus Type K Viruses Obtained from Commercial Meat Rabbits in Italy

**DOI:** 10.1128/MRA.00669-19

**Published:** 2019-10-10

**Authors:** Gianpiero Zamperin, Adelaide Milani, Alice Fusaro, Alessia Schivo, Luca Zandonà, Luca Bano, Isabella Monne

**Affiliations:** a Division of Comparative Biomedical Sciences, Istituto Zooprofilattico Sperimentale delle Venezie, Legnaro, Italy; b SCT2, Istituto Zooprofilattico Sperimentale delle Venezie, Fontane di Villorba, Italy; KU Leuven

## Abstract

Rabbit endogenous lentivirus type K (RELIK) was discovered in the genome of the European rabbit (Oryctolagus cuniculus). In our study, we present three complete genome sequences of RELIK viruses generated using a target amplification approach performed on the RNA of commercial rabbits from Italy.

## ANNOUNCEMENT

Lentiviruses (family *Retroviridae*) are known as both exogenous infectious agents and endogenous copies integrated in the genomes of several mammals, including those of the European rabbit (1), the European hare (2), the gray mouse lemur (3), Malagasy lemurs (4), the ferret (5), the Malayan colugo (6), and the cat (7). Studies on these viruses are instrumental for improving our understanding of lentivirus evolution and their interaction with the host. The only available genome of a rabbit endogenous lentivirus type K (RELIK) was reconstructed from whole-genome shotgun European rabbit sequences (1). However, no full contemporary genome sequence of lentiviruses from European rabbits is available at the NCBI.

We used a metagenomic approach to identify the viral pathogens that might be associated with epizootic rabbit enteropathy (ERE), which frequently affects European rabbits (Oryctolagus cuniculus), and to characterize the total RNA content of 10 samples collected from different organs (meseraic lymph node, thymus, colon, pylorus, and cecum) of 5 symptomatic meat rabbits sampled from 4 commercial farms in Italy. Thirty milligrams of each sample was homogenized in 600 μl of RLT buffer, using a TissueLyser II (Qiagen) with 5-mm stainless steel beads for 2 × 1-min cycles at 30 Hz. Total RNA from each specimen was extracted using the RNeasy minikit (Qiagen), according to the manufacturer’s instructions, and retrotranscribed into cDNA using random hexamers with the Maxima H minus double-stranded cDNA synthesis kit (Thermo Scientific). Libraries were prepared using the Kapa HyperPlus kit and sequenced on an Illumina NextSeq platform with the NextSeq 500/550 mid output kit v2 (2 × 150-bp paired-end [PE] mode; Illumina, San Diego, CA, USA).

Sequencing yielded, on average, 22,360,802 reads per sample, ranging from 16,546,095 to 31,234,916 reads. Raw data were filtered by removing (i) reads with more than 100 bases with a Q score below 7 and (ii) duplicated paired-end reads using an in-house python script (available at https://github.com/GianpieroZamperin/Lentivirus_Rabbit/). The remaining reads were clipped from adaptors with scythe v0.991 (https://github.com/vsbuffalo/scythe) and trimmed with sickle v1.33 (https://github.com/najoshi/sickle). Reads shorter than 80 bases or that were unpaired after previous filters were discarded. After quality filtering, we recovered 6,688,827 to 16,510,552 high-quality reads per sample. We taxonomically classified individual reads with MEGAN v6.10.8 (5).

No pathogenic agents that might be involved in the etiology of ERE were identified. However, three samples (thymus and meseraic lymph node) contained reads belonging to the Lentivirus genus (142, 47, and 117 reads). As the amount of lentivirus reads was not sufficient to characterize the complete genome, we designed 6 specific primer pairs (Table 1) on the RELIK genome reconstructed by Katzourakis et al. (1) to perform a target amplification on the RNA samples using the SuperScript III one-step reverse transcription-PCR (RT-PCR) system with Platinum *Taq* polymerase High Fidelity (Invitrogen). Library preparation was performed using the Nextera XT DNA sample preparation kit and processed on an Illumina MiSeq platform with the MiSeq reagent kit V2 nano (2 × 250-bp paired-end [PE] mode; Illumina, San Diego, CA, USA).

**TABLE 1:** Primer pairs for the target amplification approach used to obtain the rabbit lentivirus genome sequence

Primer direction	Sequence (5′→3′)	Positions^*a*^	Amplicon size (bp)
Forward	TGTTAGGGAACCATTCACGG	1–20	1,700
Reverse	CATGGCCATCTTATAGGAGG	1700–1681	
Forward	GAACCCTCTATAGAACATGG	1387–1406	2,013
Reverse	CTGTCTACCTACCCAAAGGA	3400–3381	
Forward	TAGTATCAGGGAATGGACCC	3214–3233	1,586
Reverse	TTATAGGGTGTGCCTGTGGT	4800–4781	
Forward	CGGCTAATTCTGGTAGCCAT	4580–4599	1,617
Reverse	ATAGTTCCTTCCAGTGAGCAT	6197–6177	
Forward	TTGGTGGGACCTTGGAGAG	5914–5932	1,684
Reverse	ATGTTAGCCTCAAGATGAGCG	7598–7578	
Forward	CAGATGCTCGAAGCACACAC	7378–7397	1,090
Reverse	GGTTGCCACGAAGGAAGAGT	8468–8449	

a Primer positions refer to RELIK sequence obtained *in silico* and published by Katzourakis et al. (1).

For the three sequenced samples, we produced 167,242, 224,913, and 224,949 reads per sample. Raw data were quality filtered as previously described, resulting in 146,551, 194,461, and 179,937 reads per sample, respectively. High-quality reads were mapped against the RELIK genome (8,492 bp long, 42.2% GC content, and coding for 5 proteins) by Katzourakis et al. (1) using BWA v0.7.12 (6) with standard parameters. The final coverage depth ranged from 8,138- to 10,620-fold. Variants were called with LoFreq v2.1.2 (7) and standard parameters. We obtained 1,442, 1,367, and 1,203 single-nucleotide polymorphisms (SNPs) and 64, 76, and 68 indels per sample, respectively. SNPs with a frequency lower than 50% and indels changing reading frame or with a frequency lower than 50% were filtered out. The remaining variants were used to create consensus sequences. High-quality reads were then realigned against these consensus sequences by using BWA v0.7.12 (6) with standard parameters, and the alignments were inspected with Tablet (8).

We finally produced the nearly complete genomes of three lentiviruses which have the same length (8,499 bp), have the same GC content (42.1%), and possess a sequence similarity among each other of 99.1%. They also confirm the RELIK genome organization previously reported (1) and possess sequence similarities of 97.3%, 97.7%, and 97.1% against it. The availability of these RELIK sequences can be instrumental for gaining further insights on the variability and evolutionary history of this endogenous virus.

### Data availability.

The NextSeq raw data were submitted to the NCBI Sequence Read Archive (SRA) under accession number PRJNA522038. The MiSeq raw data were submitted to the NCBI SRA under accession numbers SRR8184162, SRR8184161, and SRR8184160. The complete RELIK-like genome sequences have been deposited in GenBank under accession numbers MK182288, MK182289, and MK182290, respectively.
